# Understanding the Catalytic Determinant role of Diaphorase‐Like Subunit in Formate Dehydrogenases via Redox Couples

**DOI:** 10.1002/advs.75764

**Published:** 2026-05-19

**Authors:** Kuncheng Zhang, Weisong Liu, Hao Su, Huijuan Cui, Yuanming Wang, Zhiguang Zhu, Chun You, Lingling Zhang

**Affiliations:** ^1^ University of Chinese Academy of Sciences Beijing China; ^2^ State Key Laboratory of Engineering Biology for Low‐carbon Manufacturing Tianjin Institute of Industrial Biotechnology Chinese Academy of Sciences Tianjin China; ^3^ State Key Laboratory of Microbial Metabolism School of Life Sciences and Biotechnology Shanghai Jiao Tong University Shanghai China

**Keywords:** catalytic bias, CO_2_ reduction, formate dehydrogenases, rational design, regulatory mechanism

## Abstract

Multi‐subunit formate dehydrogenases (FDHs), which catalyze the interconversion of formate and carbon dioxide (CO_2_), have drawn increasing attention for mitigating climate change and advancing environmental protection owing to their advantages of oxygen tolerance and easy heterogenous expression. However, differently sourced multi‐subunit FDHs exhibit distinct catalytic biases, and the reasons remain unclear. On the basis of the exceptional observation of *Rhodobacter aestuarii* FDH favoring CO_2_ reduction, this study unveiled an oxidation inhibition effect in exclusively NADH/NAD^+^‐involved catalysis via kinetics analysis in terms of different redox couples. Substrate truncation positioned Fdhβ as the predominant subunit. Further studies based on structural and electrochemical insights interpreted that the slow desorption of NADH is the underlying determinant for the apparent catalytic bias. Knowledge‐based rational design helped obtain a beneficial variant, *Ra*FDH β E260Y, with a 10‐fold increased catalytic activity in CO_2_ reduction, highlighting its potential for CO_2_ biotransformation and applications in low‐carbon biomanufacturing. Eventually, bioinformatic analysis suggested that the diaphorase‐like subunits and the catalysis regulation mechanism may widely exist in living organisms for modulating the redox balance of oxidoreductases, providing new insights into metabolism and catabolism.

## Graphical abstract

A unique mechanism of catalytic bias regulated by diaphorase‐like subunit in formate dehydrogenase from *Rhodobacter aestuarii* is revealed. The diaphorase‐like subunit functions act as a biological “voltage rheostat” that controls the slow release of NADH to regulate redox balance, biasing the enzyme's catalytic preference toward CO_2_ reduction over formate oxidation.

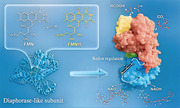

## 1 Introduction

In recent years, the alarming rise in carbon dioxide (CO_2_) concentrations has prompted the search for efficient enzymatic methods for CO_2_ fixation [1, 2, 3, 4]. Consequently, formate dehydrogenases (FDHs) have attracted considerable attention because of their catalytic characteristics in CO_2_ formate interconversion [5, 6, 7]. However, a major challenge is that most FDHs catalyze formate oxidation at a substantially higher rate. Notably, only a few FDHs exhibit catalytic bias toward CO_2_ reduction, such as those obtained from *Acetobacter woodii* (*Aw*FDH) [8], *Thermoanaerobacter kivui* (*Tk*FDH) [9], and *Rhodobacter aestuarii* (*Ra*FDH) [10]. *Aw*FDH and *Tk*FDH originate from hydrogen‐dependent CO_2_ reductases (HDCRs). *Tk*HDCR demonstrates the highest reported rate of CO_2_ reduction to date, with a *k*_cat_ value of 2654 s^−^^1^ [9]. Nevertheless, both *Aw*FDH and *Tk*FDH are oxygen‐sensitive, limiting their laboratory research and practical applications. In contrast, *Ra*FDH has been identified as an oxygen‐tolerant FDH with a three‐fold higher catalytic activity in CO_2_ reduction than formation oxidation, positioning it as a promising candidate for CO_2_ fixation [10].

*Ra*FDH is found via phylogenetic analysis based on FDHs obtained from *Rhodobacter capsulatus* (*Rc*FDH) and *Cupriavidus necator* (*Cn*FDH). *Ra*FDH exhibits a similar phylogenetic positioning and a highly similar sequence to those of *Rc*FDH (Fdhα: 89.6%, Fdhβ: 83.5%, and Fdhγ: 76.0%). In 2020, the structure of *Rc*FDH was revealed using cryo‐electron microscopy (cryo‐EM) [11]. It is a dimer of heterotetramer comprising FdsA, FdsB, FdsG, and FdsD. Early research supports that formate‐CO_2_ conversion occurs at the catalytic subunit FdsA and NAD^+^‐NADH conversion happens at the diaphorase‐like unit comprising FdsB and FdsG. In regard to catalytic biases, *Ra*FDH is distinct from *Rc*FDH. The *k*_cat_ value for formate oxidation by *Rc*FDH is 24.6 times higher (36.5 s^−1^) than for CO_2_ reduction (1.48 s^−1^) [10, 12]. However, *Ra*FDH shows a *k*_cat_ value that is 3.1 times higher for CO_2_ reduction (48.3 min^−1^) than that for formate oxidation (15.6 min^−1^). Although the cryo‐EM analysis of *Rc*FDH and its NADH‐reduced form uncovered a complex electron transfer pathway, the understanding of the catalytic modulation of formate oxidation/CO_2_ reduction bias remains insufficient. To date, limited metabolic studies have been conducted on *Rhodobacter aestuarii*, making it difficult to explain this phenomenon from a physiological perspective. Therefore, it is essential to identify the modulation factors and understand their functional mechanisms.

Multi‐subunit FDH‐catalyzed CO_2_‐formate interconversion is constituted by redox couples, such as NADH/NAD^+^, methylviologen (MV^+^/MV^2^^+^), H_2_/H^+^, and even electrodes [7], which correlate closely with catalytic thermodynamics and kinetics. NADH/NAD^+^ is by far the most common redox couple for both metal‐free and metal‐dependent FDHs. Viologen species are popular artificial redox couples that may process higher electron transfer rates than NADH/NAD^+^ [13]. FDHs from *Escherichia coli* [14, 15], *Syntrophobacter fumaroxidans* [16, 17]*, Desulfovibrio sp*. [18, 19] have been studied by using MV^+^/MV^2^^+^ for either activity determination or mediated bioelectrocatalysis. The H_2_/H^+^ couple mainly participates in the biocatalysis of HDCRs or formate–hydrogen–lyase complexes. Because some FDHs are electro‐responsive, electrodes can also work as electron donors or acceptors in FDH catalysis. The interplay and electron communication between electrodes and various FDHs have been widely studied. Therefore, investigating catalytic kinetics bias from the perspective of redox couples and studying the reaction‐induced cofactor conformation and electron transfer kinetics may provide an informative understanding of reversible biocatalysis.

Herein, *Ra*FDH‐catalyzed CO_2_‐formate interconversion was systematically examined using NADH/NAD^+^, MV^+^/MV^2^^+^, and benzyl viologen (BV^+^/BV^2^^+^) as redox couples. Catalytic kinetics studies revealed that the disparate apparent kinetics bias in NADH/NAD^+^‐involved reactions resulted from an oxidation inhibition effect. Subunit truncation of *Ra*FDH identified the diaphorase‐like Fdhβ subunit as the dominant subunit. Further investigation of electrocatalytic kinetics confirmed that enzymatic kinetics is the rate‐limiting step for the Fdhβ subunit. Analysis of the *Ra*FDH–NADH complex using cryo‐EM further elucidated the molecular mechanism, showing that the high affinity of NADH for *Ra*FDH suppressed further reaction processes. On the basis of structural understanding, the residues in the proximity to flavin mononucleotide (FMN) were rationally engineered, and a variant (*Ra*FDH βE260Y) with a 10‐fold increase in CO_2_ reduction activity was obtained. Further analyses of structure and sequence homology suggested that the FMN‐redox–induced allosteric effect may be widespread and contributes to catalytic regulation of oxidoreductases and cellular metabolic balance. The findings provide valuable insights into FDHs and other oxidoreductases involved in reversible catalysis.

## 2 Results and Discussion

### 2.1 Kinetics Characterization of *Ra*FDH Toward Formate/CO_2_ Based on Different Redox Couples

**FIGURE 1:**
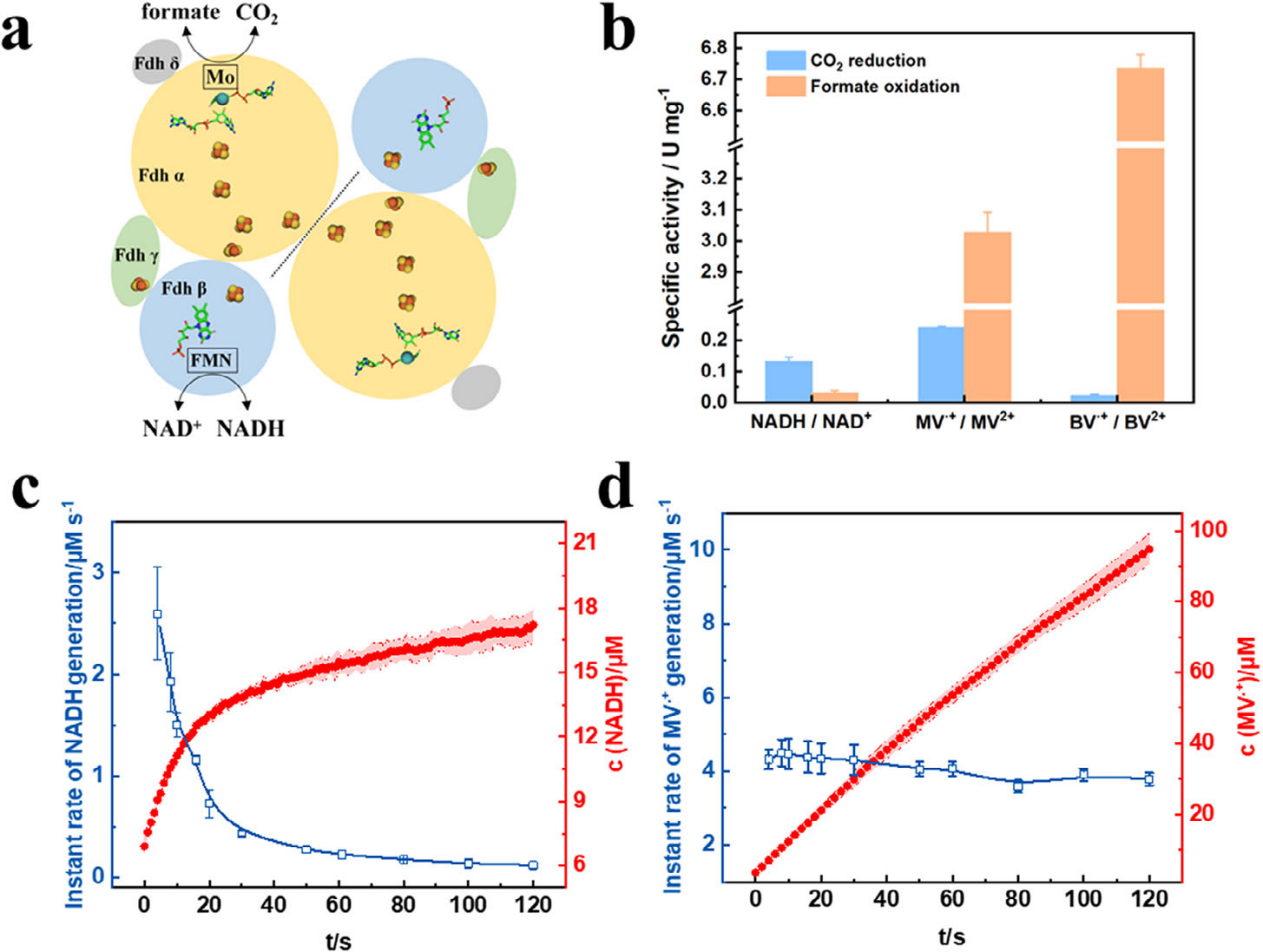
The acquisition and the kinetic performance of *Ra*FDH. (a) Schematic representation of *Ra*FDH and its cofactors (molybdenum cofactor, FMN, and iron–sulfur cluster). (b) Specific activity of *Ra*FDH for formate oxidation and CO_2_ reduction, using NADH/NAD^+^, MV^+^/MV^2+^ or BV^+^/BV^2+^ as the electron donor/acceptor. (c) NADH generation rate monitored through spectrophotometry during *Ra*FDH‐catalyzed formate oxidation. (d) MV^+^ generation rate monitored through spectrophotometry during *Ra*FDH‐catalyzed formate oxidation. All parameters were calculated from sets of three independent experiments and values reported as ± S.E.M.

*Ra*FDH was successfully expressed with a concentration of 28.7 mg L^−1^, and SDS–PAGE analysis revealed four distinct bands, corresponding to Fdhα (103.8 kDa), Fdhβ (53.0 kDa), Fdhγ (15.8 kDa), and Fdhδ (7.2 kDa) (Figure S1a). Accordingly, the schematic model of *Ra*FDH is shown in Figure 1a. After harvesting purified *Ra*FDH, the detailed enzymatic properties were measured with three redox couples, NADH/NAD^+^, MV^+^/MV^2+^, and BV^+^/BV^2+^. The specific activity for CO_2_ reduction (0.13 ± 0.02 U mg^−^^1^) was 4.1 times higher than that for formate oxidation (0.03 ± 0.01 U mg^−^^1^) when NAD^+^ (or NADH) was used (Figure 1b). These findings were consistent with previously reported results [10]. On the other hand, when MV^+^/MV^2+^ was used, increased catalytic activities were observed in both directions, confirming that the MV^+^/MV^2+^ couple possesses superior electron transfer properties to NADH/NAD^+^. However, the specific activity ratio of CO_2_ reduction to formate oxidation declined dramatically to 1:13, which further fell to 1:333 in the BV^+^/BV^2+^‐involved reaction because of the nearly diminished CO_2_ reduction activity. During the formate‐dependent kinetics determination with excess NAD^+^ as the electron acceptor, activity decreased with increasing formate concentrations (Figure S3a). At a 0.2 mM formate, the highest oxidation activity was achieved (0.43 U mg^−1^), which was 13.5 times higher than the aforementioned apparent activity (0.03 U mg^−^^1^) (Figure 1b). The catalytic inhibition at high concentrations of formate causes failure in Michaelis–Menten equation fitting, and it is reasonable to deduce that an unconventional molecular mechanism may be at play.

UV–vis spectrophotometry was employed to further investigate the *Ra*FDH kinetics catalyzing the formate–NAD^+^ reaction. As shown in Figure 1c, the introduction of *Ra*FDH resulted in a rapid decline in the NADH generation rate from 2.60 ± 0.46 to 0.43 ± 0.02 µM s^−^^1^ within the first 30 s, which differed from the steady MV^+^ generation rate in the formate–MV^2+^ reaction (Figure 1d). When *Ra*FDH was preincubated with formate for 10 min before the addition of NAD^+^ to initiate the reaction, no abrupt decrease in the NADH generation rate was observed (Figure S3b). Instead, the rate remained relatively low at 1.02 µM s^−^^1^, comparable to the post‐inhibition rate observed in the non‐preincubated experiment. Full‐wavelength scanning showed that the absorption peak of the bound FMN at 450 nm changed distinctly upon the incubation with formate, implying the reduction of FMN to FMNH_2_ (Figure S5). As the reduced state of *Ra*FDH forms before the addition of redox couples, it can be concluded that NADH/NAD^+^‐involved oxidation inhibition may be the underlying cause of catalytic bias.

### 2.2 The Catalytic Regulation of the Diaphorase‐Like Subunit of *Ra*FDH

**FIGURE 2:**
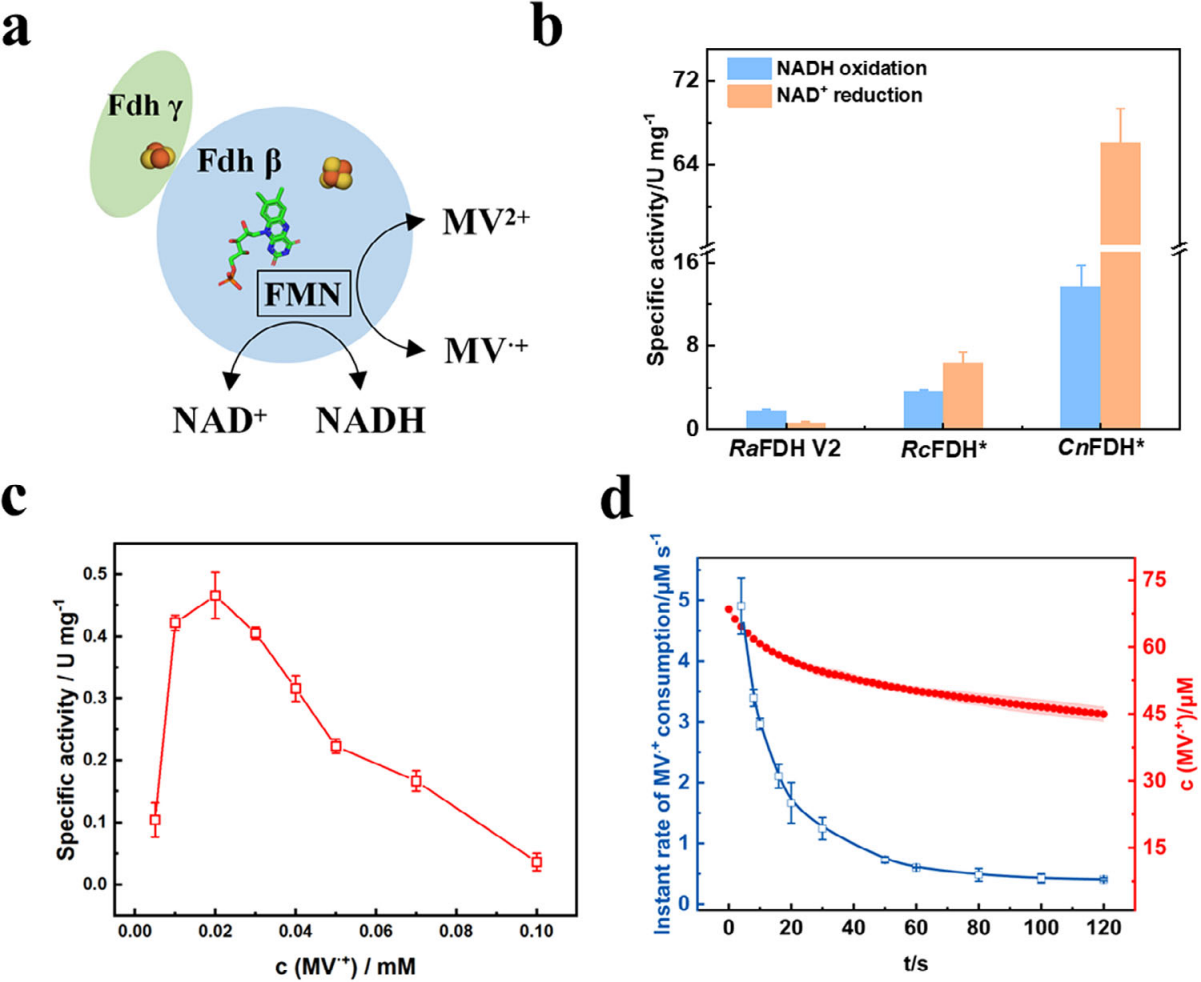
The kinetic performance of *Ra*FDH V2 variant. (a) Schematic representation of *Ra*FDH V2 (Fdhβ/γ subunits) and its cofactors (FMN and iron–sulfur cluster). (b) Specific activity of *Ra*FDH V2, *Rc*FDH*, and *Cn*FDH* for NAD^+^ reduction and NADH oxidation using MV^+^/MV^2+^ as electron donor/receptor. (c) Specific activity of *Ra*FDH V2 for NAD^+^ reduction at different MV^+^ concentrations. (d) The MV^+^ consumption rate monitored by traditional spectroscopy during *Ra*FDH V2 catalyzing NAD^+^ reduction. All parameters were calculated from sets of three independent experiments and values reported as ± S.E.M.

As no formate inhibition was observed when MV^2+^ and BV^2+^ were used as electron acceptors, it was speculated that *Ra*FDH possesses an additional electron transfer site, apart from the FMN center of the Fdhβ subunit, contributing to MV^2+^/BV^2+^‐involved formate oxidation, as indicated from a superficial iron–sulfur cluster in *Me*FDH from *Methylobacterium extorquens* AM1 [20]. In this regard, a truncated variant *Ra*FDH V1 (Fdhα/δ subunits) was obtained and investigated (Figure S1d). The activity loss in both directions (Figure S4) contradicted the above assumption, and therefore another truncated variant *Ra*FDH V2 (Fdhβ/γ subunits) was studied (Figure 2a). For further understanding, *Rc*FDH and *Cn*FDH were also truncated to leave FdsB and FdsG subunits, generating the variants *Rc*FDH* and *Cn*FDH* as controls. As shown in Figure 2b, *Ra*FDH V2, as well as the similarly truncated *Rc*FDH* and *Cn*FDH* variants, was capable of catalyzing the redox reactions of NADH‐MV^2^^+^ and MV^+^‐NAD^+^, but the catalytic bias toward CO_2_ reduction and the oxidation inhibition by high concentrations of MV^+^ were only observed in the case of *Ra*FDH V2 (Figure 2c,d), indicating that *Ra*Fdhβ is the predominant subunit to cause catalytic bias and that its interaction with NAD^+^ may adopt an unfavorable conformation. In principle, electrons derived from the oxidation of formate or MV^+^ by *Ra*FDH or *Ra*FDH V2 flow to the Fdhβ subunit, transiting the catalytic active site from an electron‐deficient oxidized state, FMN, to an electron‐rich reduced state, FMNH_2_. A notable structural difference exists between NAD^+^ and MV^2+^. NAD^+^ relies on the nicotinamide ring to undertake electron communication with FMNH_2_, which requires a favorable stacking conformation as well as a sufficient electron transfer distance. In comparison, smaller MV^2+^ molecules interact with FMNH_2_ via its conjugated structure, increasing the contact frequency and making electron transfer more flexible. Regarding the above deduction, more molecular‐level evidence is needed.

### 2.3 Understanding Molecular Mechanisms from an Electrochemical Perspective

**FIGURE 3:**
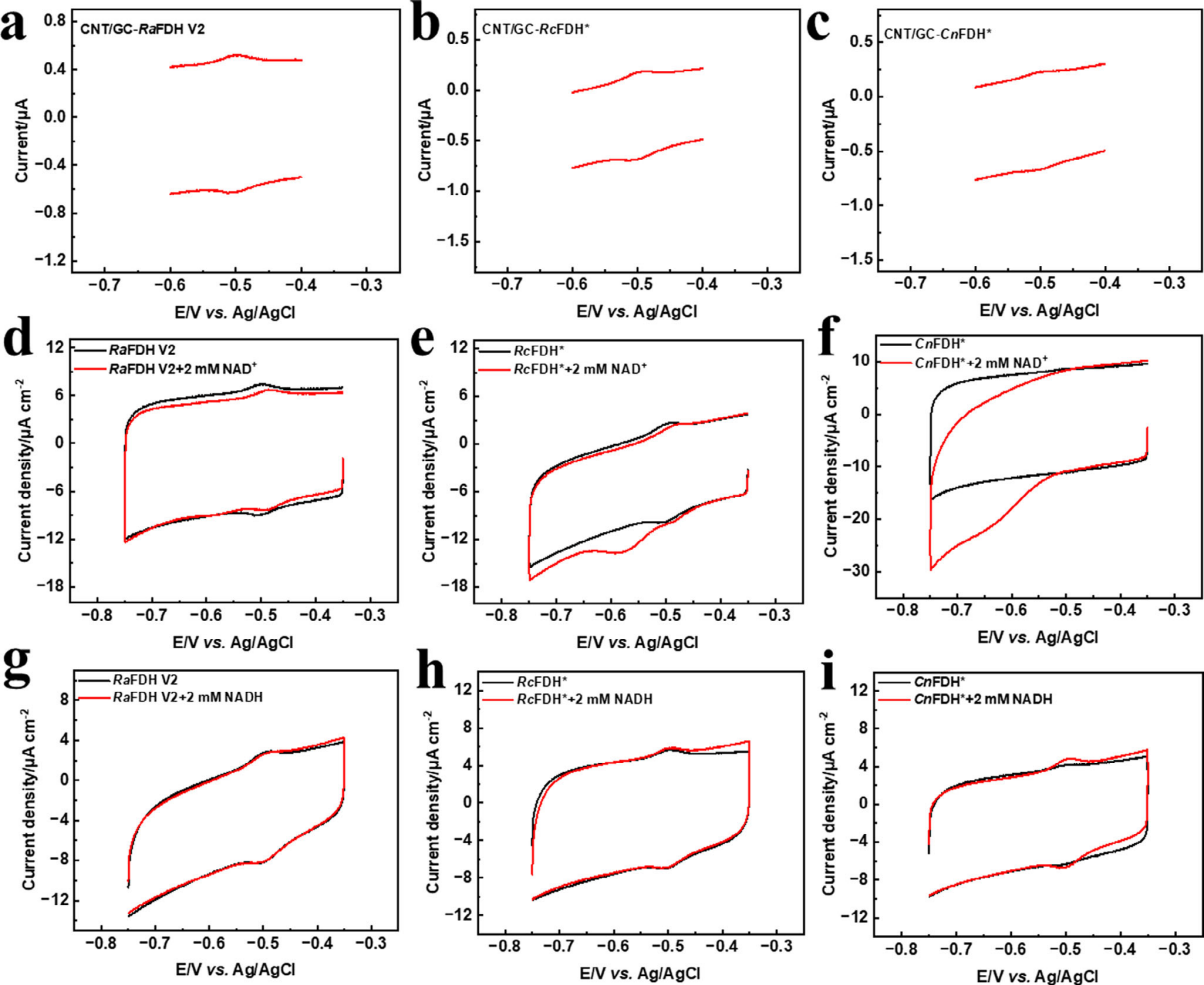
Electrochemical characterizations of truncated variants. Electrochemical analysis of (a) *Ra*FDH V2, (b) *Rc*FDH*, and (c) *Cn*FDH*. (d) Bioelectrocatalytic NAD^+^ reduction by *Ra*FDH V2. (e) Bioelectrocatalytic NAD^+^ reduction by *Rc*FDH*. (f) Bioelectrocatalytic NAD^+^ reduction by *Cn*FDH*. (g) Bioelectrocatalytic NADH oxidation by *Ra*FDH*. (h) Bioelectrocatalytic NADH oxidation by *Rc*FDH*. (i) Bioelectrocatalytic NADH oxidation by *Cn*FDH*. The scan rate was 10 mV s^−1^, the buffer was 0.1 M K_2_PO_4_, pH 7.5, and the temperature was 25°C.

Electrochemical techniques provide a powerful approach to study the catalytic sites of oxidoreductases and the electron/proton transfer in redox catalysis. *Ra*FDH V2, as well as the similarly truncated *Rc*FDH* and *Cn*FDH* variants, were immobilized onto carbon nanotube–modified glassy carbon electrodes, and protein film voltammetry (PFV) was carried out to characterize the redox peaks of the FMN cofactor and the electrocatalytic performance toward NADH and NAD^+^. As shown in Figure 3a–c, redox peaks assigned to FMN were observed in all three truncated variants, even though the formal potentials (*E^0‵^*) and the peak intensities showed slight differences. The *E^0‵^* of *Ra*FDH V2 was −0.51 V vs. Ag/AgCl, while the *E^0‵^* of *Rc*FDH* and *Cn*FDH* were −0.50 V vs. Ag/AgCl. Regarding electrocatalysis, truncated *Rc*FDH* and *Cn*FDH* showed higher catalytic current toward NAD^+^ reduction than *Ra*FDH V2 (Figure 3d–f), with the highest current density (13.7 µA cm^−2^) observed at the truncated *Cn*FDH* electrode; this finding aligned with the specific activities of full enzymes in formate oxidation, suggesting that the enzymatic kinetics, rather than interfacial electron transfer or mass transfer, is the rate‐limiting step in the electrocatalytic process. Another notable observation was that all three of these variants exhibited little catalytic current in NADH oxidation (Figure 3g–i); nevertheless, *Ra*FDH exhibited three‐fold higher catalytic activity in CO_2_ reduction than in formate oxidation. Generally, a typical electrochemical process contains at least five steps: diffusion to the electrode surface, adsorption, chemical reaction with electron transfer, desorption, and product removal. Following this pattern, it is deduced that the subtle current may be attributable to either collapsed catalytic kinetics or sluggish desorption. Collapsed catalytic kinetics was verified by the aforementioned oxidation inhibition effect, and the much lower *K*_m_ value of NADH than that of NAD^+^ (Table 1) may suggest a sluggish desorption of the formed NADH during NAD^+^ reduction.

**TABLE 1:** Steady‐state kinetics (*K*_m_, *k*_cat_, and *k*_cat_/*K*_m_) of *Ra*FDH and *Ra*FDHV2.

Enzymes	Substrate	Electron donor/acceptor	*K*_m_ (mM)	*k*_cat_ (s^−1^)	*K*_cat_/*K*_m_ (s^−1^ mM^−1^)
*Ra*FDH	HCOOH	NAD^+^	N.D.	N.D.	N.D.
	HCO_3_^−^	NADH	5.97 ± 1.97	0.20 ± 0.02	0.03 ± 0.01
	HCOOH	MV^2+^	0.28 ± 0.02	12.64 ± 0.17	44.44 ± 0.60
	HCO_3_^−^	MV^+^	0.19 ± 0.05	2.61 ± 0.10	13.96 ± 0.05
*Ra*FDH V2	MV^+^	NAD^+^	N.D.	N.D.	N.D.
	NAD^+^	MV^+^	N.D.	N.D.	N.D.
	MV^2+^	NADH	3.19 ± 0.37	0.77 ± 0.05	0.24 ± 0.02
	NADH	MV^2+^	0.02± 0.01	0.56 ± 0.01	29.42 ± 0.34

All parameters were calculated from sets of three independent experiments and the results were reported as ± S.E.M. Michaelis–Menten plots are shown in Figure S2. N.D. indicates that the value was not detectable; when NAD^+^ was used as the substrate, the Michaelis–Menten equation for *Ra*FDH at different formate concentrations and MV^+^ did not fit well, as the activity of *Ra*FDH decreased with the increasing concentration of formate and MV^+^.

### 2.4 Understanding Molecular Mechanisms from a Structural Perspective

**FIGURE 4:**
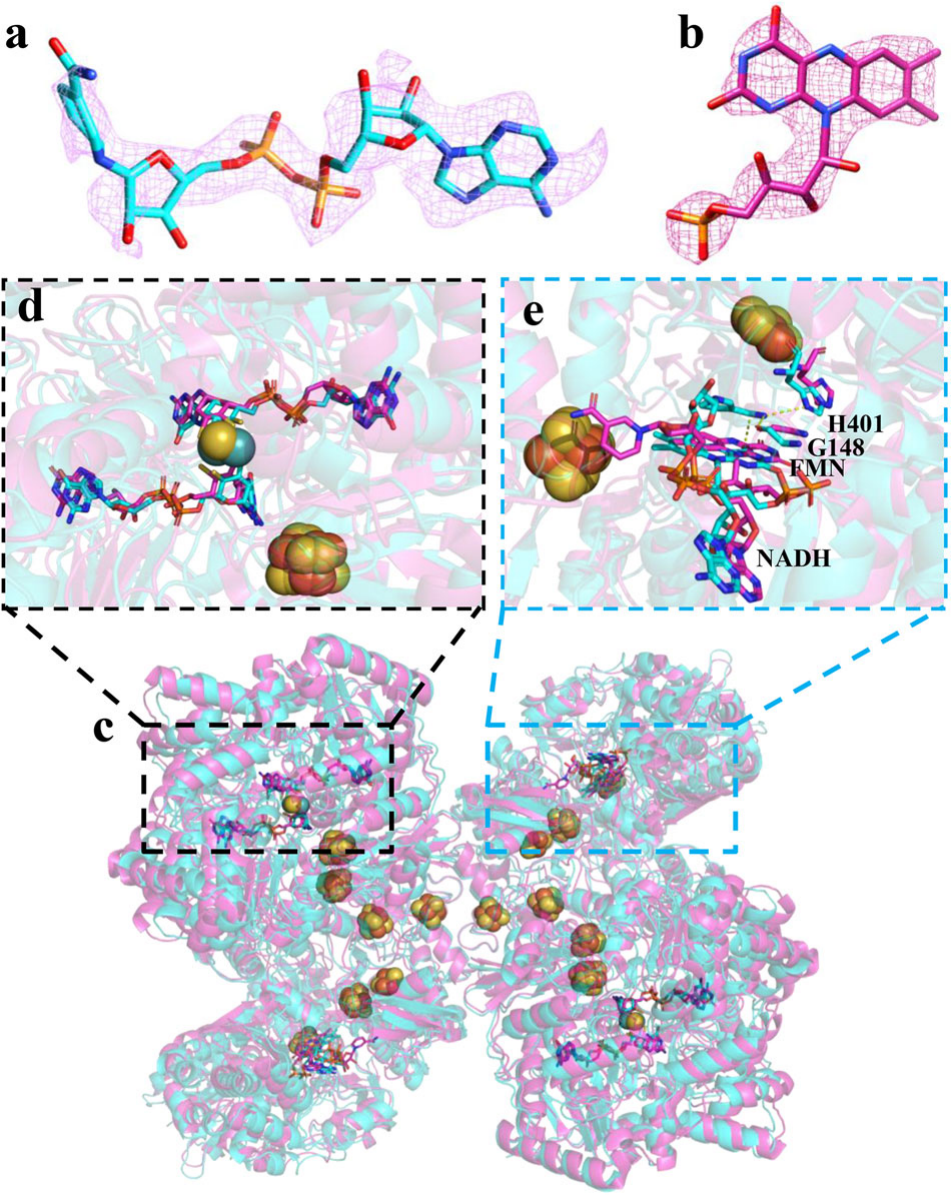
Structure analysis of *Ra*FDH. (a) Electron density map of NADH. (b) Electron density map of FMN. (c) Active center in Fdhα. (d) Active center in Fdhβ. (e) Structure analysis by aligning *Ra*FDH (cyan) and reduced *Rc*FDH (PDB 6tg9) (wine red).

Sequence alignment revealed a high identity of 83.5% between the *Ra*FDH β subunit and *Rc*FDH, while both showed ∼60.7% identity with *Cn*FDH (Figure S6). No significant difference was observed between the sequences. To elucidate the molecular mechanism underlying catalytic bias, the structure of the *Ra*FDH‐NADH complex was resolved using cryo‐EM, yielding a final reconstruction with an overall resolution of 2.9 Å (Figure S7). Similar to *Rc*FDH, *Ra*FDH is a dimer of heterotetramer comprising four subunits, Fdhα, Fdhβ, Fdhγ, and Fdhδ. Subunit Fdhα harbors a bis‐molybdopterin guanine dinucleotide (bis‐MGD) cofactor, subunit Fdhβ loads an FMN cofactor and binds NADH (Figure 4a,b); seven Fe‐S clusters are distributed in proximity to bis‐MGD and FMN. After aligning the resolved structure of the *Ra*FDH–NADH complex (PDB: 9WXB) with the reported isolated *Rc*FDH structure (PDB: 6TG9), no obvious differences were observed in the overall protein shells (Figure 4c) or the main catalytic Fdhα subunit (Figure 4d), with a root mean square deviation of 0.6 Å, validating that Fdhα is not the predominant subunit influencing the catalytic bias. Structural differences were observed in subunit Fdhβ (Figure 4e). In *Ra*FDH, the nicotinamide ring forms a tight π–π stacking interaction with the isoalloxazine ring of FMN and hydrogen bonds with H401. In contrast, the nicotinamide moiety of *Rc*FDH is located in front of the FMN‐binding pocket, and the distance between the C4 in the nicotinamide ring of NADH and the C10 in the piperazine ring of FMN is as much as 11.6 Å, not facilitating efficient electron transfer. Moreover, no effective hydrogen bonds form between NADH and the surrounding residues to stabilize the catalytic conformation, leading to a decreased affinity of *Rc*FDH for NADH. This observation can also explain the apparent *K*_m_ value difference between *Ra*FDH and *Rc*FDH. The *K*_m_ value of *Rc*FDH for NADH was undetectable, and that for NAD^+^ was 0.173 mM [12]. The *K*_m_ value of *Ra*FDH was 0.02 mM for NADH (Table 1) but was undetectable for NAD^+^, indicating that *Ra*FDH possesses a higher affinity for NADH. The formate inhibition effect observed in *Ra*FDH is likely caused by NADH molecules generated during formate oxidation that fail to desorb from the Fdhβ subunit because of strong affinity, thereby slowing the subsequent reaction rate. Accordingly, the catalytic bias of *Ra*FDH is attributed to its markedly higher affinity for NADH.

To further reveal the potential auxiliary factors related to the electron transfer efficiency of different redox couples (MV^2+^, BV^2+^), we performed molecular docking of the *Ra*FDH β subunit with MV^2+^ and BV^2+^, respectively. As shown in Figure S8, in the lowest‐energy conformation of the *Ra*FDH β subunit with MV^2+^ and BV^2+^, neither MV^2+^ nor BV^2+^ forms additional interactions with the subunit, apart from the π–π stacking interaction between BV^2+^ and FMN. This contrasts with the hydrogen bonds and electrostatic interactions observed between NADH and *Ra*FDH β subunit, suggesting that the binding forces for MV^2+^ and BV^2+^ are weak.

Additionally, the *K*_m_ value of *Ra*FDH V2 for MV^2+^ is 3.19 mM, which is 159.5‐fold higher than that for NADH (0.02 mM) (Table 1), indicating a low affinity to MV^2+^. Meanwhile, a slow dissociation process was not observed during the kinetics determination of MV^2+^‐participated reaction, which is definitely different from NAD^+^‐involved catalysis Furthermore, the distances between MV^2+^/BV^2+^ and the B6 [4Fe‐4S] cluster are both 6.8 Å, suggesting a direct and rapid electron shuttling in between, without requiring the relay of FMN. In contrast, FMN is indispensable for NADH‐involved catalysis and in this case, MV^2+^ and BV^2+^ may facilitate the electron transfer process. This is further supported by the observation that the apparent catalytic activity of WT is substantially higher with MV^2+^/BV^2+^ as electron acceptors than that with NAD^+^ as electron acceptor. Therefore, the diaphorase‐like β subunit modulates catalytic preference in a manner that depends on NAD^+^/NADH.

*Ra*FDH might be much promising in the practical biocatalytic applications, especially for the autotrophic metabolic engineering of common heterotrophic organisms, due to its unique preference for CO_2_ reduction. In most cases, integration of formate dehydrogenases plays the role of oxidative dissimilation to generate reducing equivalents in *vivo* [21, 22]. For instance, *Cn*FDH was integrated into *Escherichia coli* to improve the growth on formate. However, *Cn*FDH favors formate oxidation and its primary function is to oxidize formate to regenerate NADH [23]. In contrast, the integration of *Ra*FDH may strengthen the carbon assimilation due to its unique preference for CO_2_ reduction. In addition, its superiorities of oxygen tolerance, abundant expression in common chassis like *E. coli* endow it the universal versatility in CO_2_ biotransformation and low‐carbon biomanufacturing.

**FIGURE 5:**
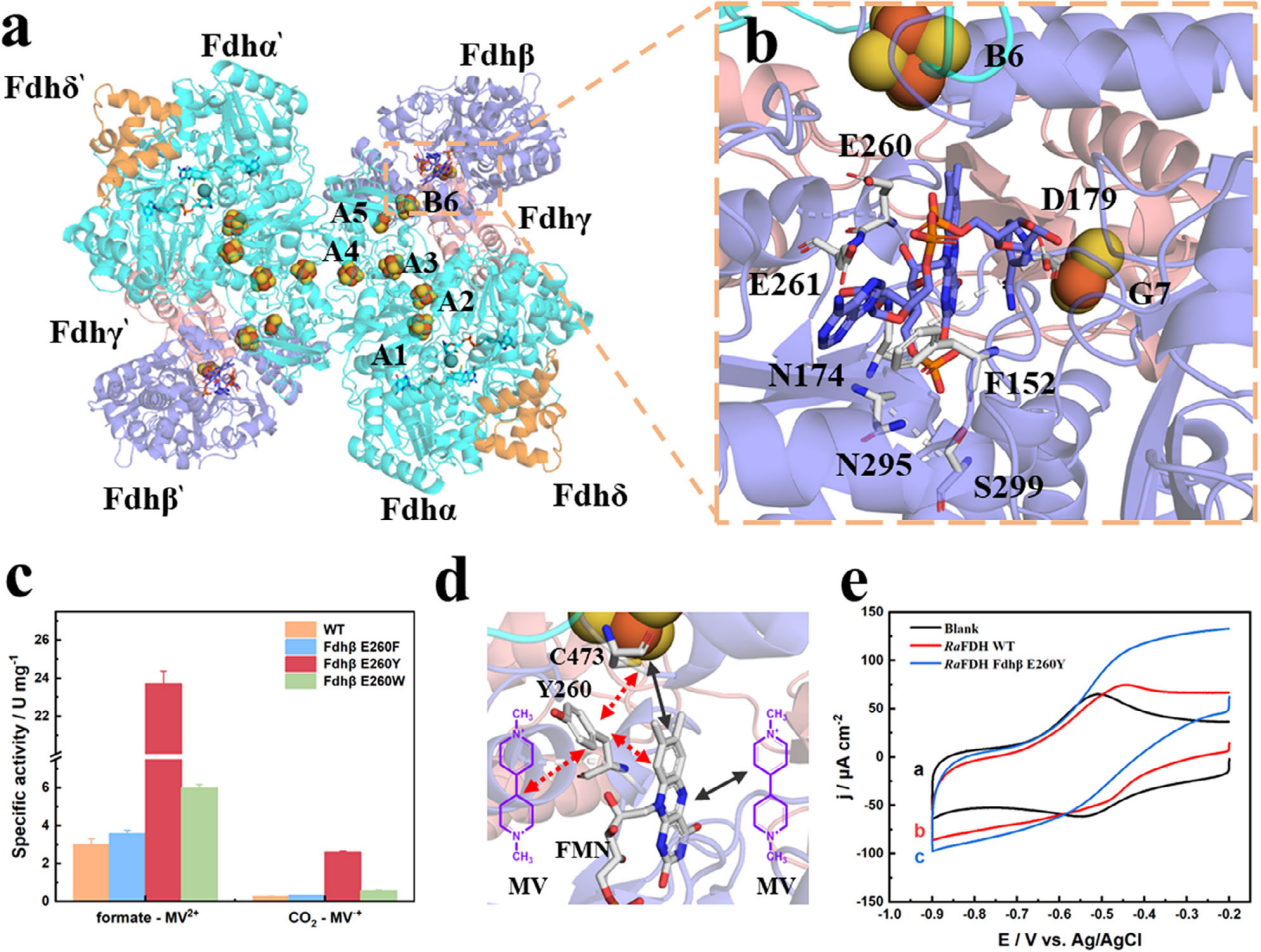
Engineering *Ra*FDH to improve catalytic activity. (a) Overview structure of *Ra*FDH. (b) Hotspot selected within β subunit. (c) Catalytic activity of *Ra*FDH and its variants using methyl viologen (MV) as the electron donor/acceptor. (d) Proposed electron transfer pathway in E260Y variant. (e) Bioelectrocatalytic CO_2_ reduction and formate oxidation by *Ra*FDH and its E260Y variant, 20 mM HCO_3_^−^ and 20 mM formate as the substrate. The scan rate was 10 mV s^−1^ at 0.1 M K_2_PO_4_, pH7.5.

Despite the catalytic bias of *Ra*FDH to CO_2_ reduction, the insufficient *k*_cat_ value impedes the possible practical applications in CO_2_ biotransformation. According to the obtained structural and mechanistic understanding, the residues between FMN and the B6 [4Fe‐4S] cluster were analyzed and found to be mainly composed of aliphatic amino acids (E260, D179, N174 and E261) (Figure 5b). While aromatic amino acids, owing to their conjugated structures, are the primary electron transfer mediators in proteins [24]. For instance, phenylalanine has been found to play a crucial role in the efficient electron transfer between carbon monoxide dehydrogenase and viologen [25]. The catalytic activity of cytochrome P450s was improved 13.6‐fold through introducing aromatic amino acids to enhance electron transfer rate [26]. Recently, the direct electron transfer capability of formate dehydrogenase from *Shewallena oneidensis* MR‐1 was unveiled to be attributed to the abundant aromatic amino acids (tyrosine and phenylalanine) around the enzyme surface [27]. Thus, the aliphatic amino acids between FMN and the B6 [4Fe‐4S] cluster were substituted by aromatic amino acid considering their “conductive” electron‐conjugated structures.

The activity of variants was screened, and it found that substitutions at position E260 in Fdhβ by phenylalanine, tyrosine, and tryptophan were selected owing to their increased specific activities toward MV^+^/MV^2^^+^ involved catalysis (Figures S9 and S10). Among them, *Ra*FDH βE260Y exhibited the highest catalytic performance, with a 7.83‐fold increase in oxidized activity (23.71 ± 0.68 U mg^−^^1^) and a 10.73‐fold increase in reductive activity (2.59 ± 0.08 U mg^−^^1^) compared to those of wild‐type *Ra*FDH (*Ra*FDH WT) (Figure 5c). As per the structural insights (Figure 5d), the substitution of tyrosine bridges FMN and the B6 [4Fe‐4S] cluster facilitating the electron transfer. Additionally, the similar electron‐conjugated molecular structures between tyrosine and methyl viologen may have promoted the rapid electron communication to the MV^+^/MV^2+^ redox couple. In the subsequent electrocatalysis characterization of *Ra*FDH WT and *Ra*FDH βE260Y, both the oxidation current and reduction current increased at the *Ra*FDH βE260Y electrode (Figure 5e). In addition, the *Ra*FDH βE260Y electrocatalysis MV^2+^ reduction rate was higher than that of *Ra*FDH WT (Figure S11), further validating the accelerated electron transfer to MV^+^/MV^2+^ redox couple.

The Fdhβ subunit of *Ra*FDH catalyzes the interconversion of NADH and NAD^+^, most prevalent reactions in physiological metabolism. A systematic sequence–structure–function analysis was performed using bioinformatics techniques. The sequence of the Fdhβ subunit was found to be highly conserved in nature. Enzymes such as hydrogenases and respiratory complex I display a high degree of sequence‐related and structural similarity in the diaphorase‐like subunit (Figure S12), although the homology among other subunits in these enzymes differs. The most predominant conserved loop (S‐G‐L‐R‐G‐R‐G‐G‐A‐G‐F‐P‐T‐G), features six glycine residues out of the 14 residues, which is generally considered to favor the flexibility of the FMN‐catalytic pocket. Shomura et al. revealed that the reduced Ni‐Fe hydrogenase by H_2_ undergoes allosteric regulation within its flavin‐binding subunit in the absence of a physiological electron acceptor, leading to release of its FMN cofactor and consequent loss of activity [28]. Similarly, FMN dissociation was also observed in the oxidation state of *Escherichia coli* respiratory complex I [29]. This mechanism protects the enzyme from oxygen poisoning by preventing the transfer of electrons to oxygen, which would generate damaging reactive oxygen species (ROS). However, we revealed that FMN remains fully bound in reduced *Ra*FDH (Figure 4b), with catalytic activity primarily regulated by slow dissociation of NADH, representing a distinct regulatory strategy. This regulatory mechanism may help maintain intracellular redox balance and prevent carbon loss due to formate oxidation. The opposite catalytic preferences of highly homologous *Ra*FDH and *Rc*FDH may reflect the distinct intracellular demands of carbon and energy metabolism. *Rc*FDH originates from *Rhodobacter capsulatus*, a bacterium that fixes CO_2_ via the Calvin–Benson–Bassham (CBB) cycle rather than through formate dehydrogenases [30, 31]. Thus, *Rc*FDH may primarily function in formate oxidation to generate NADH and detoxify formic acid [32]. While *Ra*FDH is thought to function in CO_2_ reduction within the cell, likely through regulation of CO_2_ preference by a diaphorase like subunit to prevent carbon loss via formate oxidation. The role of the regulatory mechanism in *Ra*FDH in cellular metabolism requires further investigation.

## 3 Conclusions

This study primarily revealed an oxidation inhibition‐induced apparent catalytic bias in NADH/NAD^+^‐involved *Ra*FDH catalyzing CO_2_‐formate interconversion. Kinetics characterization demonstrated that the catalytic bias in favor of CO_2_ reduction occurs exclusively in the presence of the NADH/NAD^+^couple; this is because the reaction in the oxidation direction is suppressed at formate concentrations higher than 0.2 mM. When viologen species were applied as the redox couples, the oxidation activity of *Ra*FDH significantly exceeded its reduction activity, as observed in most FDHs. Investigations of truncated *Ra*FDH V2 using enzymatic and electrocatalytic kinetics indicated that the Fdhβ subunit serves as the regulatory subunit responsible for this inhibition. Further structural analysis revealed that the sluggish desorption of NADH is the underlying determinant of the apparent catalytic bias. Based on structural and mechanistic understanding, a *Ra*FDH variant (βE260Y) was rationally designed and obtained with improved catalytic activities in both directions, providing promising biocatalysts for CO_2_ biotransformation. Moreover, it was found that diaphorase‐like subunits and catalytic regulation may represent a widespread mechanism in nature, modulating the redox balance of oxidoreductases.

## 4 Material and Methods

### 4.1 Reagents and Materials

All chemicals used in this study were of analytical grade or higher purity and were sourced from Sigma–Aldrich, Macklin (Shanghai, China), and Sinopharm (Beijing, China), unless otherwise specified. Primers and genes were synthesized by Tsingke (Beijing, China). Phanta Max Super DNA Polymerase and a ClonExpress‐II one‐step cloning kit were purchased from Vazyme (Nanjing, China). The methylation‐sensitive restriction enzyme *Dpn*I was obtained from New England Biolabs. Lysogeny Broth (LB) medium (1 L) was prepared by adding 10 g of NaCl, 10 g of tryptone, and 5 g of yeast extract. For LB agar plates, 20 g of agar was added, and all media were autoclaved at 121°C for 20 min.

### 4.2 Plasmid Construction

The gene cluster sequence containing Fdhγ, Fdhβ, Fdhα, Fdhδ, and FdhD from *Rhodobacter aestuarii* was synthesized and cloned into the pTrc‐HisA plasmid, following the protocol described in a previous study [10]. A His‐tag was added to the N‐terminus to facilitate purification. The plasmid encoding the truncated *Ra*FDH variants was generated by a one‐step PCR method. Site‐directed mutagenesis was performed using the QuikChange method, with the mutation sites introduced by primers during PCR. The polymerase chain reaction (PCR) mixture (50 µL) contained 25 µL of Phanta Max Super DNA Polymerase, 20 ng of the pTrc‐HisA‐*Ra*FDH template, and 500 nM of forward (F) and reverse (R) primers (The primers sequence was shown in Table S1). The PCR cycling conditions were as follows: initial denaturation at 95°C for 5 min, followed by 25 cycles of denaturation at 95°C for 30 s, annealing at 55°C for 30 s, and extension at 72°C for 5 min, with a final extension at 72°C for 10 min. The PCR products were analyzed by agarose gel electrophoresis, and methylated templates were specifically degraded by *Dpn*I. The PCR products were purified using a DNA purification kit (TIANGEN, Beijing, China) and subsequently ligated using the ClonExpress‐II one‐step cloning kit (37°C for 30 min). The ligation products were then transformed into 100 µL of E. coli Top10 competent cells (heat‐shocked at 42°C for 45 s) and recovered at 37°C for 30 min. The transformed cells were plated on LB agar plates containing 50 mg L^−^^1^ ampicillin and incubated overnight at 37°C. Colonies were selected from the LB agar plates and cultured in LB medium with 50 mg L^−^^1^ ampicillin for sequencing verification and storage. The gene sequence was confirmed by Tsingke (Beijing, China).

### 4.3 Expression and Purification

Overexpression of *Ra*FDH was achieved by transforming the pTrc‐HisA‐*Ra*FDH plasmid into *E. coli* MC1061 cells. The cells were initially precultured in 10 mL of LB medium containing 50 µg mL^−1^ ampicillin at 37°C, 220 rpm overnight. The preculture was then transferred into 1 L of LB medium with 1 mM sodium molybdate and the same concentrations of ampicillin, and the culture was grown at 37°C, 220 rpm, until the OD600 reached 0.60–0.80. Overexpression was induced by adding IPTG (final concentration 0.2 mM) and ferric ammonium citrate (final concentration 0.5 mM). The culture was then incubated at 25°C, 150 rpm, for 24 h. After incubation, cells were harvested by centrifugation and resuspended in 100 mM potassium phosphate buffer (pH 7.5) containing 10 mM sodium nitrate. Cell lysis was carried out using a high‐pressure homogenizer, and the resulting cell debris was removed by ultracentrifugation (12,000 rpm, 4°C). The supernatant containing the soluble protein was loaded onto a Ni‐NTA affinity column via gravity flow. To remove non‐specific proteins, the column was washed with 25 mM and 50 mM imidazole, each containing 10 mM sodium nitrate. The target protein was eluted with 400 mM imidazole in the same buffer. The eluted protein was concentrated and further purified by size‐exclusion chromatography on a Superdex 200 column using AKTA protein purification system. Through the UV detector of the system, each protein peak was analyzed by SDS‐PAGE to verify the *Ra*FDH peak and its purity. The protein concentration was measured according to the Bradford method. The proteins were separated on 15% polyacrylamide gels and stained with Coomassie brilliant blue G250. The *Ra*FDH‐containing peak fractions were pooled, concentrated, filtered, and immediately used for kinetic characterization or cryo‐EM sample preparation.

### 4.4 Enzyme Activity Assay

Enzyme activity assays were performed within an anaerobic chamber. For *Ra*FDH and its engineered variant (βE260), Buffer A (100 mM HPO_4_^2^^−^/H_2_PO_4_^−^, 10 mM NaNO_3_, pH 7.5) was employed.

Reaction using NADH/NAD^+^ as a substrate: For formate oxidation, the reaction mixture contained 20 mM sodium formate (substrate) and 5 mM NAD^+^, with the total volume adjusted to 2 mL. Absorbance at 340 nm (NADH, ε = 6.22 mM^−^^1^ cm^−^^1^) was monitored using an Evolution One spectrophotometer (Thermo Fisher Scientific) equipped with a temperature control module, following the addition of *Ra*FDH and its variants. One unit of oxidation activity was defined as the oxidation of 1 µmol of formate (reducing 1 µmol of NAD^+^) per minute under the assay conditions. For CO_2_ reduction, the reaction mixture contained 20 mM sodium formate (substrate) and 0.16 mM NADH, with the total volume also adjusted to 2 mL. The decrease in absorbance at 340 nm (NADH) was monitored as described above. One unit of reduction activity was defined as the reduction of 1 µmol of CO_2_ (oxidizing 1 µmol of NADH) per minute under the assay conditions.

Reaction using MV as a substrate: For formate oxidation, the reaction mixture contained 20 mM sodium formate (substrate) and 5 mM MV^2^^+^ (electron acceptor), with the total volume adjusted to 2 mL. Absorbance at 604 nm (MV^+^, ε = 13.9 mM^−^^1^ cm^−^^1^) was monitored using an Evolution One spectrophotometer (Thermo Fisher Scientific) following the addition of *Ra*FDH and its variants. One unit of oxidation activity was defined as the oxidation of 1 µmol of formate (reducing 2 µmol of MV^2^^+^) per minute under the assay conditions. For CO_2_ reduction, the reaction mixture contained 20 mM sodium formate (substrate) and 5 mM MV^2^^+^, with the total volume adjusted to 2 mL. For CO_2_ reduction using MV^+^, MV^2^^+^ was first reduced by sodium dithionite (DTH) to generate reduced MV^+^, which served as the electron donor for CO_2_ reduction. The decrease in absorbance at 604 nm (MV^+^) was monitored as described above. One unit of reduction activity was defined as the reduction of 1 µmol of CO_2_ (oxidizing 2 µmol of MV^+^) per minute under the assay conditions.

The reaction substrates for the *Ra*FDH truncated variants (Fdhβ/γ subunits) are NADH and MV^2^^+^, or NAD^+^ and MV^+^. For enzyme activity assays, Buffer A (100 mM HPO_4_^2−^/H_2_PO_4_^−^, 10 mM NaNO_3_, pH 7.5) was utilized. For the reaction oxidizing NADH to produce MV^+^, the reaction mixture contained 5 mM NADH (substrate) and 5 mM MV^2^^+^, with the total volume adjusted to 2 mL. Absorbance at 604 nm (MV^+^, ε = 13.9 mM^−^^1^ cm^−^^1^) was monitored using an Evolution One spectrophotometer (Thermo Fisher Scientific) following the addition of *Ra*FDH variants. One unit of oxidation activity was defined as the oxidation of 1 µmol of NADH (reducing 2 µmol of MV^2^^+^) per minute under the assay conditions. For the reaction oxidizing MV^+^ to produce NADH, MV^2^^+^ was first reduced by sodium dithionite (DTH) to generate reduced MV^+^, which served as the electron donor for NAD^+^ reduction. The reaction mixture contained 0.1 mM MV^+^ (substrate) and 5 mM NAD^+^, with the total volume also adjusted to 2 mL. The decrease in absorbance at 604 nm (MV^+^) was monitored as described above. One unit of reduction activity was defined as the oxidation of 2 µmol of MV^+^ (reducing 1 µmol of NAD^+^) per minute under the assay conditions.

### 4.5 Kinetic Characterization

Kinetic parameter assays were conducted in Buffer A (100 mM HPO_4_^2−^/H_2_PO_4_^−^, 10 mM NaNO_3_, pH 7.5) within an anaerobic chamber. The reaction system consisted of substrate A (formate/HCO_3_^−^) and substrate B (NADH/NAD^+^, MV^+^/MV^2^^+^) in Buffer A. For both formate oxidation and CO_2_ reduction, excess substrate (other than the one being tested) were added to ensure substrate saturation. *Ra*FDH or its variants was introduced into the reaction system to initiate the reaction. Absorbance changes were measured at varying concentrations of the substrate/product (NADH at 340 nm and MV^+^ at 604 nm) following the addition of *Ra*FDH and its variants. The absorbance changes were converted to corresponding substrate consumption or product formation rates (µmol min^−^^1^) and fitted to the Michaelis–Menten model using Origin software. Each dataset represents the average of n = 3 independent experiments, with error bars indicating the standard error of the mean (S.E.M.).

### 4.6 Full‐Wavelength Absorbance Scans

Full‐wavelength UV–visible absorbance scans were performed to determine whether *Ra*FDH could catalyze reactions in the presence of a single substrate (formate or NADH) using an Evolution One spectrophotometer (Thermo Fisher Scientific). FMN exhibits an absorption peak at 450 nm, while the peak disappears when it is reduced to FMNH_2_. Standard control assays were conducted in Buffer A (100 mM HPO_4_^2−^/H_2_PO_4_^−^, 10 mM NaNO_3_, pH 7.5) with 5 mg mL^−1^ *Ra*FDH within an anaerobic chamber. Formate (final concentration: 20 mM) or NADH (final concentration: 5 mM) was added to the system and incubated for 10 min before full‐wavelength scanning was performed. The scan data were processed using Origin software.

### 4.7 Transient Kinetic Analysis

All experiment was performed using an Evolution One spectrophotometer (Thermo Fisher Scientific) in an anaerobic chamber. For the transient kinetic detection of *Ra*FDH for formate oxidation using NAD^+^ as electron accept, the reaction was initiated by adding 10 µg *Ra*FDH to a Buffer A solution containing 20 mM formate and 5 mM NAD^+^, and the absorption changes were recorded at 340 nm (ε = 6.22 mM^−1^ cm^−1^). For the transient kinetic detection of *Ra*FDH for formate oxidation using methylviologen as electron accept, the reaction was initiated by adding 10 µg *Ra*FDH to a Buffer A solution containing 20 mM formate and 5 mM MV^2+^, and the absorption changes were recorded at 604 nm (MV^+^, ε = 13.9 mM^−^^1^ cm^−^^1^). For the transient kinetic detection of *Ra*FDH V2, the reaction was initiated by adding 10 µg *Ra*FDH V2 to a Buffer A solution containing 5 mM NAD^+^ and 0.1 mM reduced methylviologen, and the absorption changes were recorded at 604 nm (MV^+^, ε = 13.9 mM^−^^1^ cm^−^^1^).

### 4.8 Electrochemical Analysis

The electrochemical analysis was performed by potentiostat (CHI660e) in an anaerobic glove box. A three‐electrode system was used, consisting of a glassy carbon electrode (0.07 cm^2^) as the working electrode, a platinum wire electrode the counter electrode, and an Ag/AgCl electrode as the reference electrode. The glassy carbon electrode was polished with an alumina slurry (0.05 µm) on a polishing cloth, then sonicated three times for 1 min each in deionized water and once in ethanol. The Carbon nanotube (CNT) was dispersed in water and dimethylformamide (DMF) (ratio of 1:1) to a concentration of 1 g L^−1^. A CNT/GC electrode was constructed by drop‐casting 2 µL CNT dispersion onto a polished GC electrode, with drying at 60°C. Both bioelectrocatalytic and electrochemical tests were performed by adding 0.2 g L^−1^ enzymes (*Ra*FDH V2, *Rc*FDH*, *Cn*FDH* and *Ra*FDH‐β/γ‐E260Y). All experiments were applied at 0.1 m K_2_PO_4_, pH 7.5.

### 4.9 Cryo‐EM Analysis

The protein peak corresponding to freshly purified *Ra*FDH was selected for negative‐staining electron microscopy (EM) experiments to assess the aggregation behavior, homogeneity, and orientation of the protein in solution. The results showed that *Ra*FDH did not aggregate and exhibited uniformity with no apparent preferred orientation. Subsequently, freshly purified protein was applied onto holey‐carbon cryo‐EM grids at a concentration of 1 mg ml^−1^, vitrified in liquid ethane using a Vitrobot, and stored in liquid nitrogen until further use. The protein was incubated with 1 mM formate for 10 min, followed by a 20‐min incubation with NADH at a 1:20 ratio prior to vitrification. Cryo‐EM data were collected at the Center for Cryo‐Electron Microscopy at Zhejiang University. Micrographs were acquired on a Titan Krios microscope (FEI) operating at 300 kV, equipped with a Selectris energy filter (Thermo Fisher Scientific) and a Gatan Falcon 4 detector. Automated data collection was performed using EPU software according to standard protocols. A calibrated magnification of ×130 000 was used, corresponding to a pixel size of 0.93 Å. The defocus range was set between −0.8 and −1.5 µm. Each micrograph was dose‐fractionated into 40 frames under a dose rate of 7.49 e^−^ per pixel per second, with a total exposure time of 6 s, resulting in a cumulative dose of approximately 50 e^−^ Å^−2^.

For image processing, MotionCor2 [33] was used for motion correction and dose‐weighting of frames, while CTFFind4.1 [34] was employed to estimate the contrast transfer function (CTF) parameters from the movie frames. Frames exhibiting contamination or poor CTF estimation were discarded. All subsequent image processing steps were performed using RELION 3.1 [35]. A total of 5,679 micrographs were collected, and 5 906 502 particles were auto‐picked and extracted with a binning factor of 3 for 3D classification. Selected particles were re‐extracted to a pixel size of 0.93 Å for 3D refinement with C2 symmetry and Bayesian polishing. The final 3D reconstruction, derived from 192 652 particles, yielded an EM map with a resolution of 2.90 Å.

The structure model was subsequently built and refined against the electron density using COOT [36]. Atomic models were iteratively refined through real‐space refinement cycles in the PHENIX software package [37], incorporating secondary structure constraints, and were then manually adjusted in COOT. Molecular structures and their corresponding densities were visualized and presented using ChimeraX [38] and PYMOL. The obtained density maps and model files have been deposited in the Protein Data Bank (PDB) and Electron Microscopy Data Bank (EMDB), and the PDB ID is 9WXB and the EMDB ID is EMD‐66342.

### 4.10 Molecular Docking Analysis

The structural mode of *Ra*FDH V2 was used for docking with Methyl viologen (MV) and benzyl viologen (BV). The molecular structures of MV and BV were minimized using Chem3D with the MM2 force field. Molecular docking was performed by Autodock4. The box size was set to 50 × 40 × 40 points in the x, y, and z dimensions, respectively, and the box center was set to coordinates of (149.378, 103.765, 129.749) in the x, y, and z dimensions, respectively.

## Funding

This study was supported by Coal‐Major Project (2025ZD1701600), Tianjin Major Science and Technology Projects and Engineering Programs (National Key Laboratory Projects) (25ZXZSSS00010), Strategic Priority Research Program of the Chinese Academy of Sciences (XDC0120103), the Beijing‐Tianjin‐Hebei Natural Science Foundation Cooperation Project (NO. 25JJJJC0036).

## Conflicts of Interest

None of the authors have a conflict of interest to disclose.

## Supporting information




**Supporting file**: advs75764‐sup‐0001‐SuppMat.docx

## Data Availability

The data that support the findings of this study are available from the corresponding author upon reasonable request.
