# Supplementary material for: Understanding the Catalytic Determinant role of Diaphorase‐Like Subunit in Formate Dehydrogenases via Redox Couples
Source: Adv Sci (Weinh). 2026 May 19:e75764. Online ahead of print. doi: 10.1002/advs.75764 (PMC13335904; doi:10.1002/advs.75764)
Supplement: Supplementary file 1 — Supporting file: advs75764‐sup‐0001‐SuppMat.docx [file ADVS-9999-e75764-s001.docx]

Supporting Information

Understanding the Catalytic Determinant role of Diaphorase-Like Subunit in Formate Dehydrogenases via Redox Couples

Kuncheng Zhang^1, 2, ＃^, Weisong Liu^1, 2, ＃^, Hao Su^1, 2^, Huijuan Cui^2^, Yuanming Wang^2^, Zhiguang Zhu^1, 2^, Chun You^3^, Lingling Zhang ^1, 2, *^

^1^ University of Chinese Academy of Sciences, Beijing 100049, China

^2^ State Key Laboratory of Engineering Biology for Low-carbon Manufacturing, Tianjin Institute of Industrial Biotechnology, Chinese Academy of Sciences, Tianjin 300308, China

^3^ State Key Laboratory of Microbial Metabolism, School of Life Sciences and Biotechnology, Shanghai Jiao Tong University, Shanghai 200240, China

^＃^ These authors contribute equally to this work.
E-mail: [zhangll@tib.cas.cn](mailto:zhangll@tib.cas.cn)

**
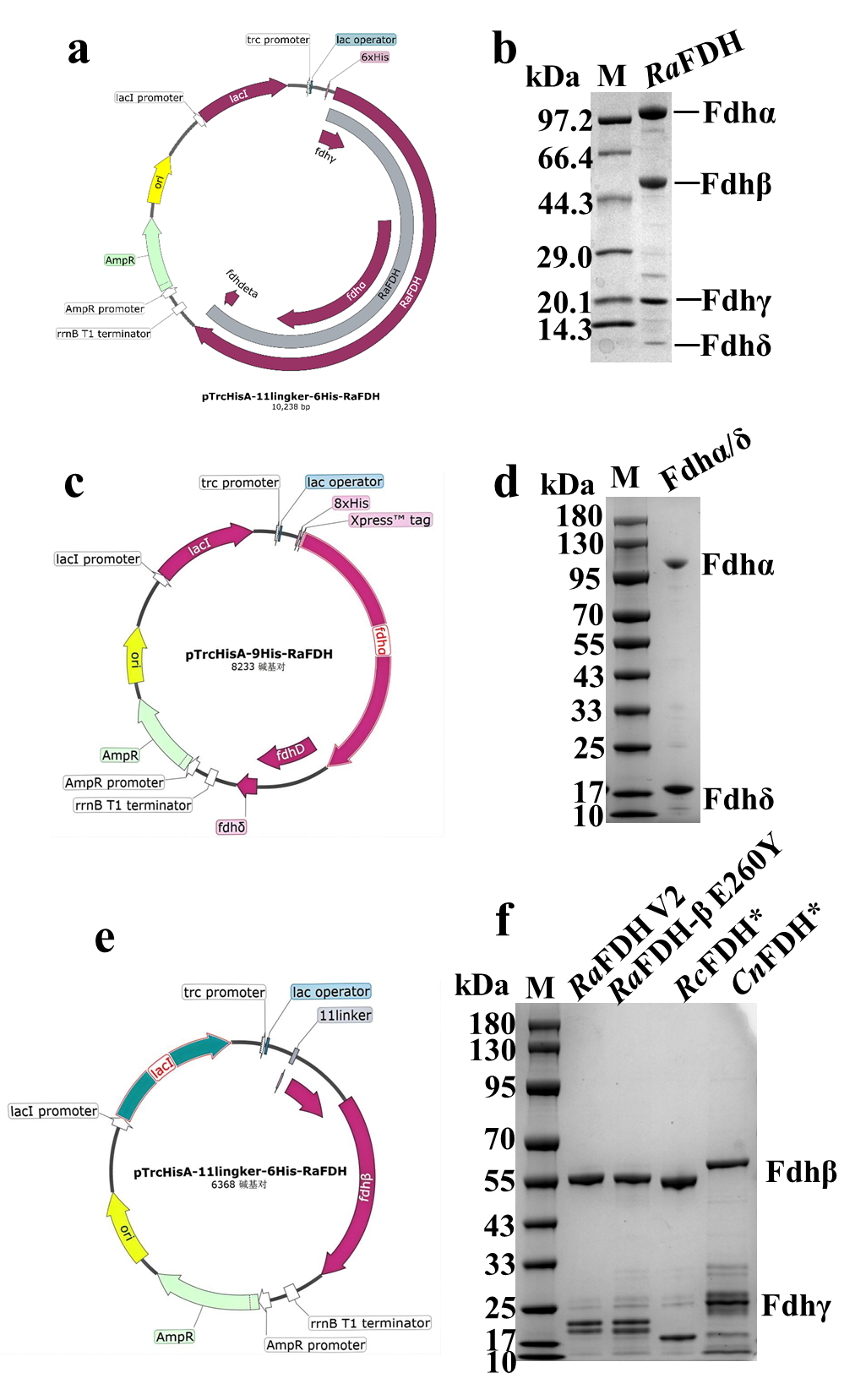
**Figure S1. (a) pTrc-HisA-*Ra*FDH was used to express *Ra*FDH (Fdhα, Fdhβ, Fdhγ, Fdhδ and Fdh D), (b) SDS-PAGE analysis of the purified *Ra*FDH expressed in *E. coli* MC1061, (c) pTrc-HisA-Fdhα/δ plasmid used for the expression of *Ra*FDH V1, (d) SDS-PAGE analysis of the purified *Ra*FDH V1, (e) pTrc-HisA-Fdhβ/γ plasmid used for the expression of Fdhβ/γ of *Ra*FDH V2, *Rc*FDH* and *Cn*FDH*, (f) SDS-PAGE analysis of the purified Fdhβ/γ.


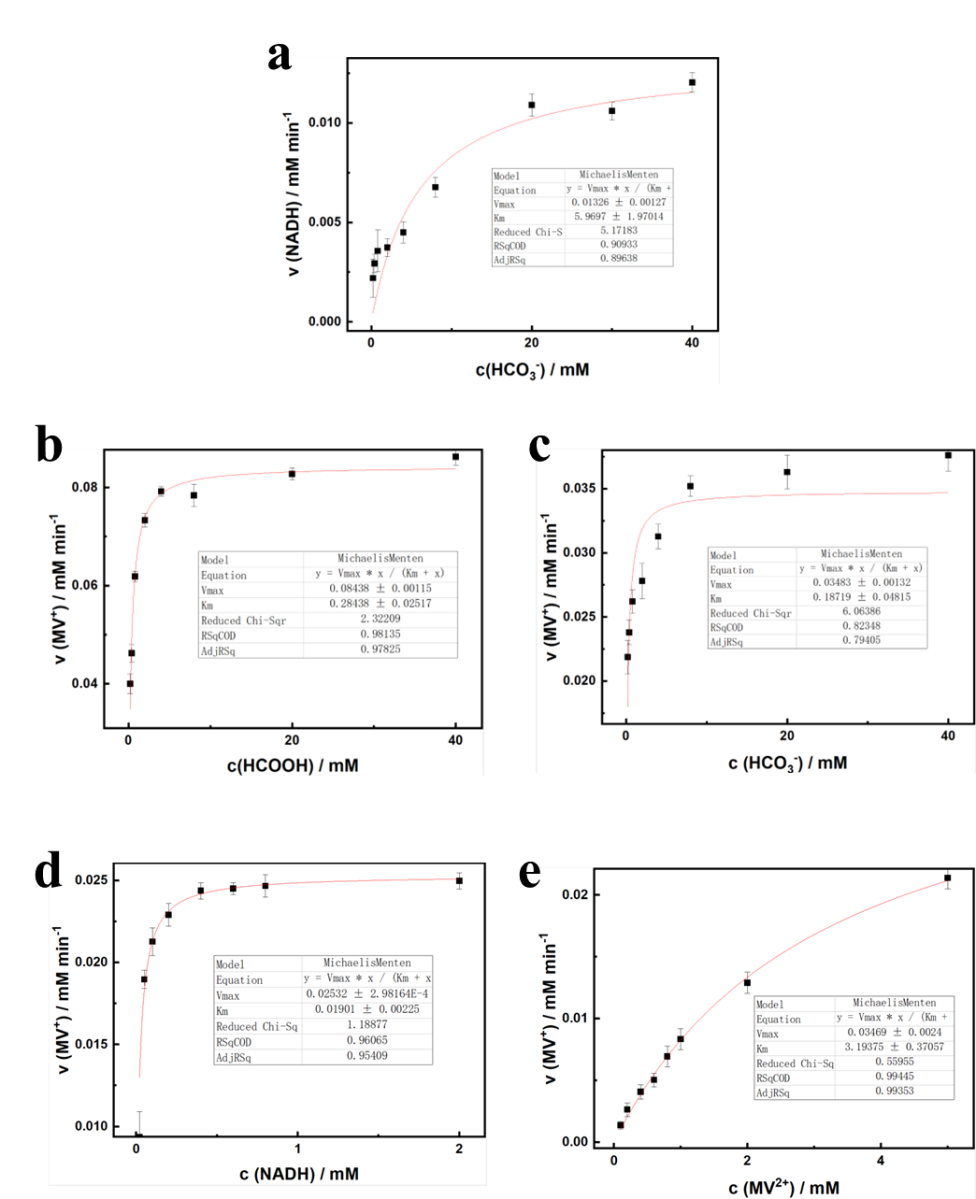
Figure S2. Steady-state kinetic analysis of *Ra*FDH and *Ra*FDH V2, Steady-state kinetic assays were performed by monitoring the change in absorbance at 340 nm for NADH (ε = 6.22 mM⁻¹ cm⁻¹) and at 604 nm for MV^·+^ (ε = 13.9 mM⁻¹ cm⁻¹), as previously described and outlined in the Materials & Methods section. Michaelis-Menten plots for *Ra*FDH were generated by varying the concentrations of the following substrates: (a) HCO_3_^-^ using NADH as the substrate (ranging from 0.2 to 40.0 mM), (b) formate using MV^2^⁺ as the substrate (ranging from 0.2 to 40.0 mM), (c) HCO_3_^-^ using MV^2^⁺ as the substrate (ranging from 0.2 to 40.0 mM), (d) Michaelis-Menten plots for *Ra*FDH-Fdhβ/γ were constructed by varying the concentrations of NADH (ranging from 0.002 to 2.0 mM) and (e) MV²⁺ (ranging from 0.1 to 5 mM). The Michaelis-Menten plot for *Ra*FDH with varying concentrations of formate using NAD^+^ as the substrate could not be properly fitted, likely due to a decrease in enzyme activity at higher formic acid concentrations. The kinetic parameters derived from these experiments are summarized in Table 1. Each dataset represents the average of n = 3 independent experiments, with error bars indicating the standard error of the mean (S.E.M.).


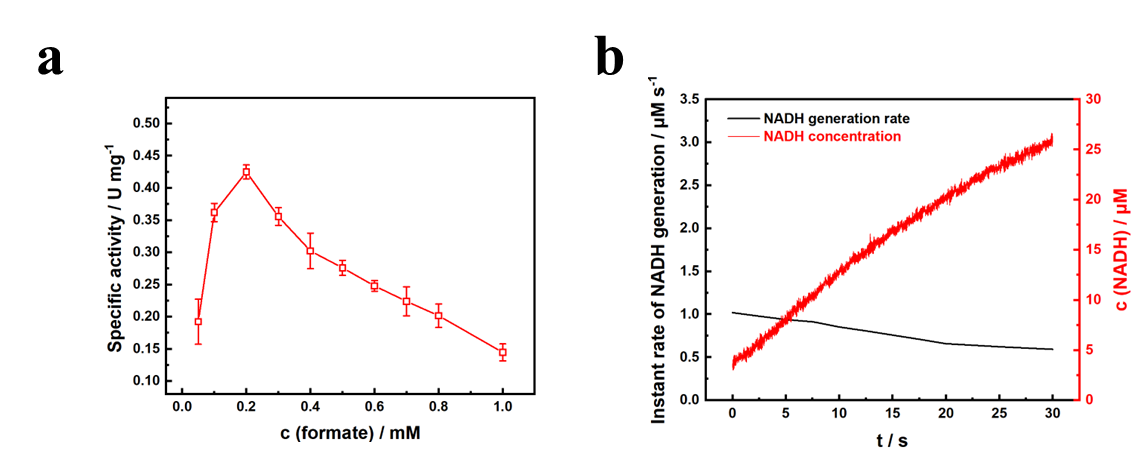
Figure S3. Transient kinetic analysis of formate inhibition. (a) Specific activity of *Ra*FDH at different formate concentrations using NAD^+^ as electron acceptor, (b) NADH generation rate monitored by spectrophotometry during *Ra*FDH catalyzed formate oxidation after pre-incubation with formate.


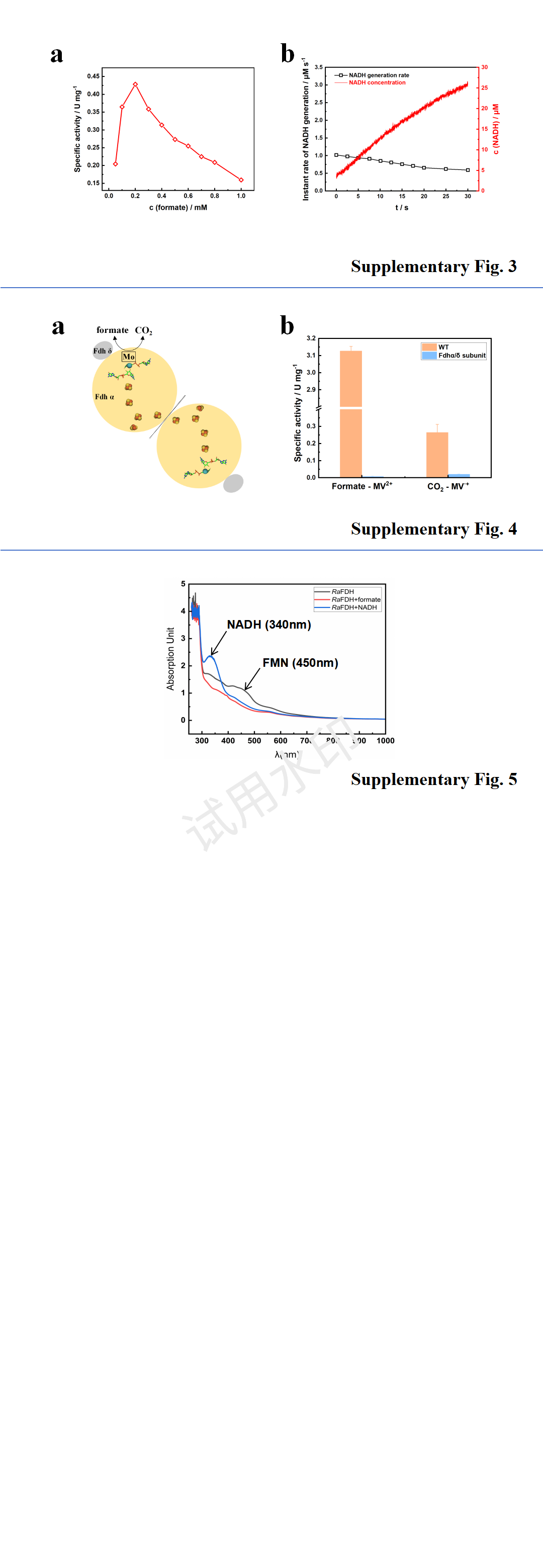


Figure S4. (a) Schematic representation of the *Ra*FDH V1 complex (Fdhα/δ subunits), highlighting the MoCo and iron-sulfur cluster, (b) Specific activity of *Ra*FDH V1 and *Ra*FDH in formate oxidation and CO_2_ reduction, measured with methylviologen as the electron donor/acceptor.


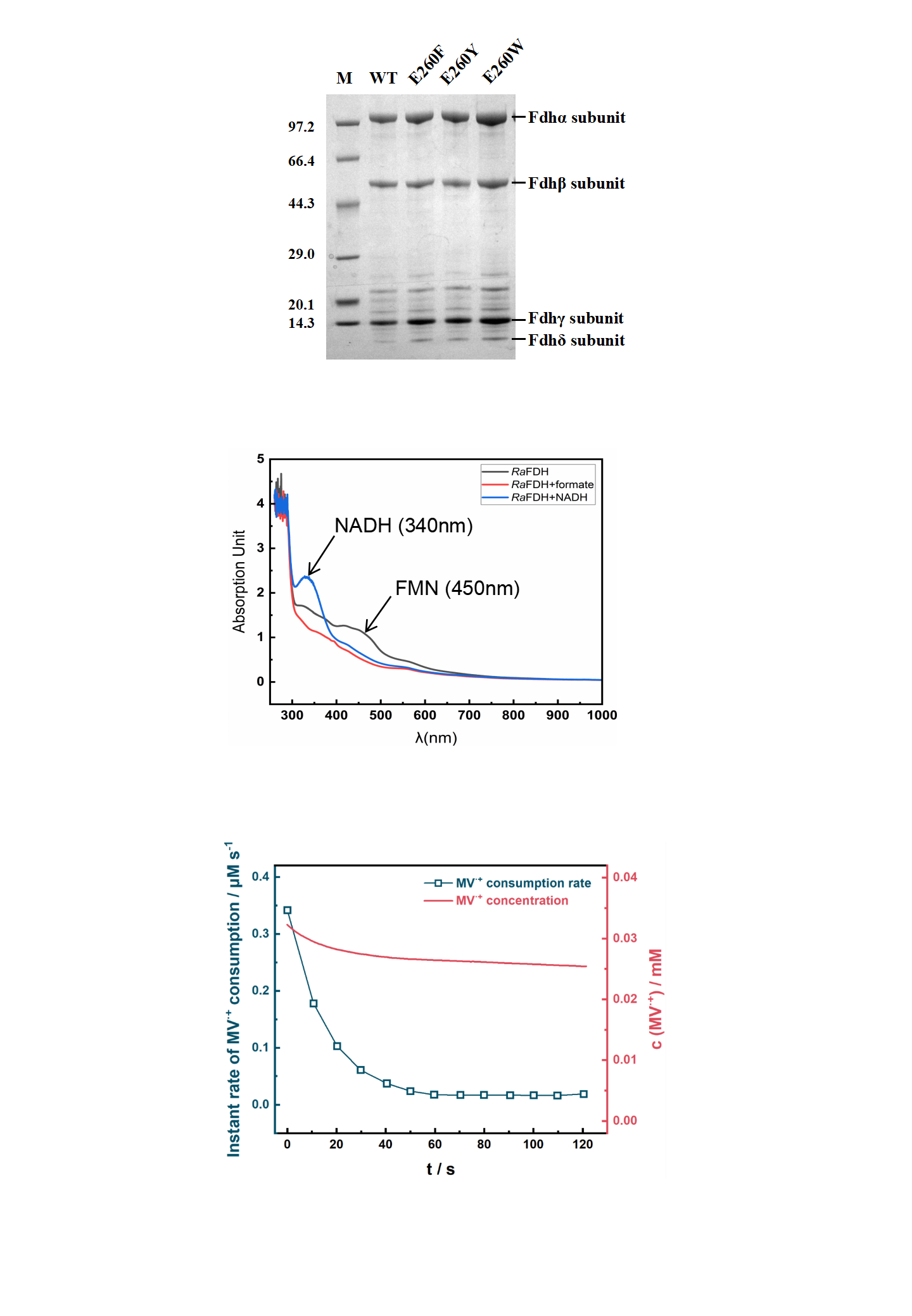


Figure S5. Full-wavelength scans of *Ra*FDH were conducted in the presence of various substrates. The reactions were performed in a buffer containing 100 mM HPO_4_^2^⁻/H_2_PO_4_⁻ and 10 mM NaNO_3_ at pH 7.5, with the following conditions: no substrate (black), 20 mM formate (red), or 5 mM NADH (blue). Absorption peaks for NADH were observed at 340 nm, while FMN absorption was detected at 450 nm.


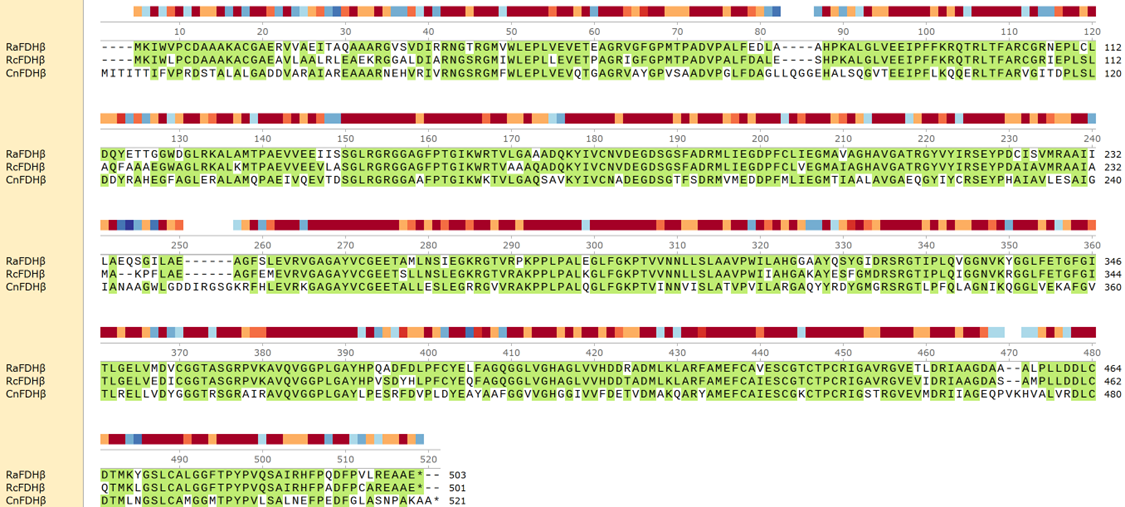


Figure S6. Sequence identity analysis of *Ra*FDH β, *Rc*FDH β and *Cn*FDH β subunit.

**
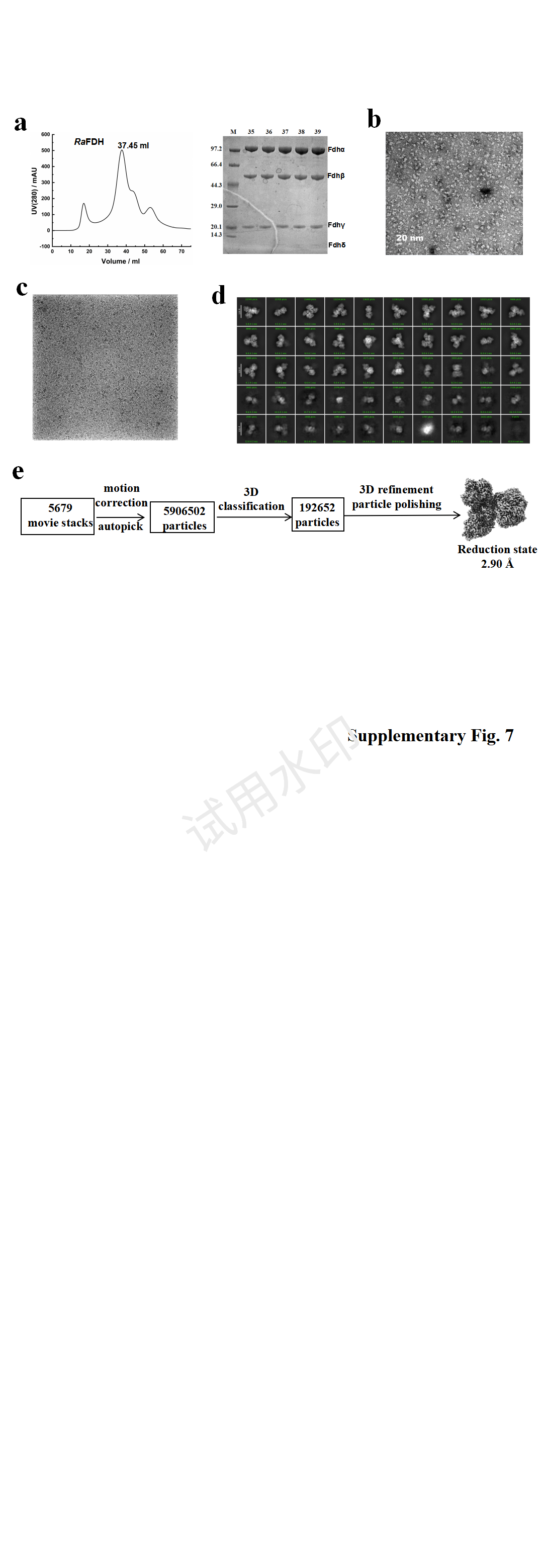
**

Figure S7. Structure determination of *Ra*FDH in reduced States, (a) Size-exclusion chromatography using a Superdex 200 column was performed, followed by SDS-PAGE analysis to assess the purity of *Ra*FDH. (b) Negative staining was applied to the purified *Ra*FDH to evaluate the sample quality. (c) Representative micrographs of *Ra*FDH in reduced states are presented, providing visual evidence of the structural integrity, (d) 2D classification results for *Ra*FDH in the reduced states are shown, highlighting particle alignment and classification quality, (e) Flowcharts of the image processing pipelines for the reduced state *Ra*FDH are provided, illustrating the steps taken to refine the particle images.


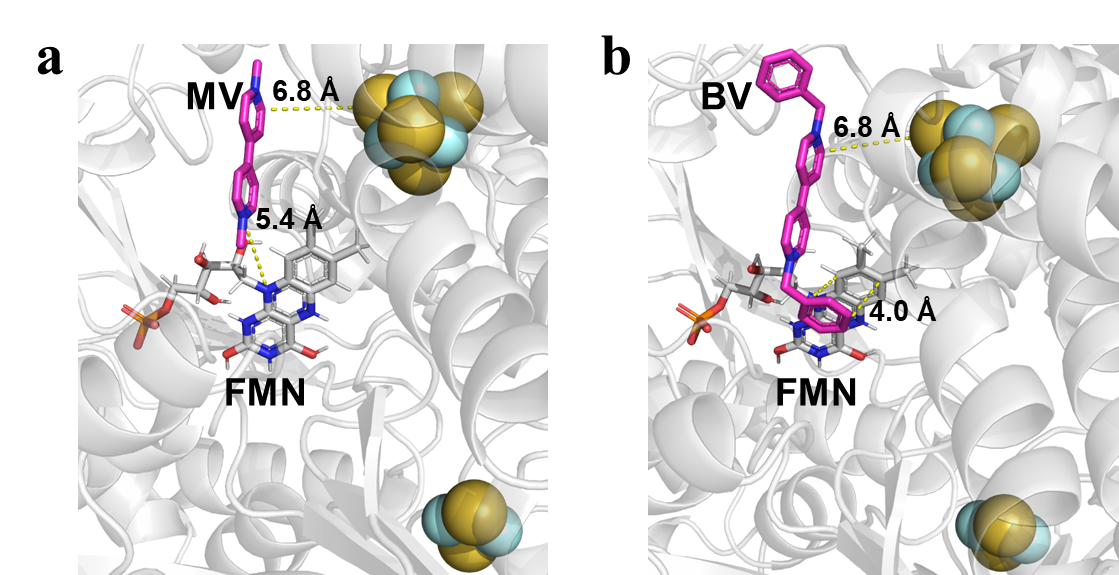
Figure S8. The docking analysis of *Ra*FDH β subunit with MV^2+^ and BV^2+^. (a) The docking conformation of *Ra*FDH β subunit with MV^2+^, (b) The docking conformation of *Ra*FDH β subunit with BV^2+^.


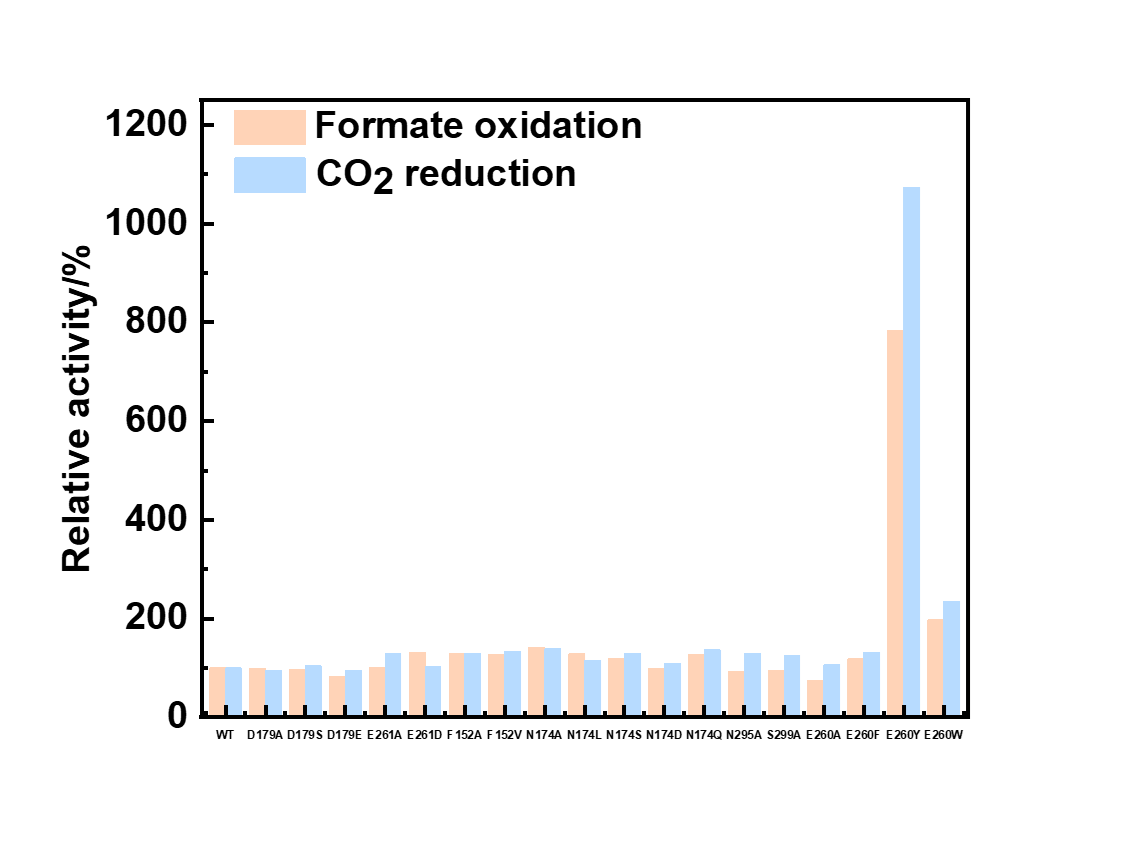


Figure S9. The relative activity of *Ra*FDH and it’s variants for CO_2_ reduction and formate.


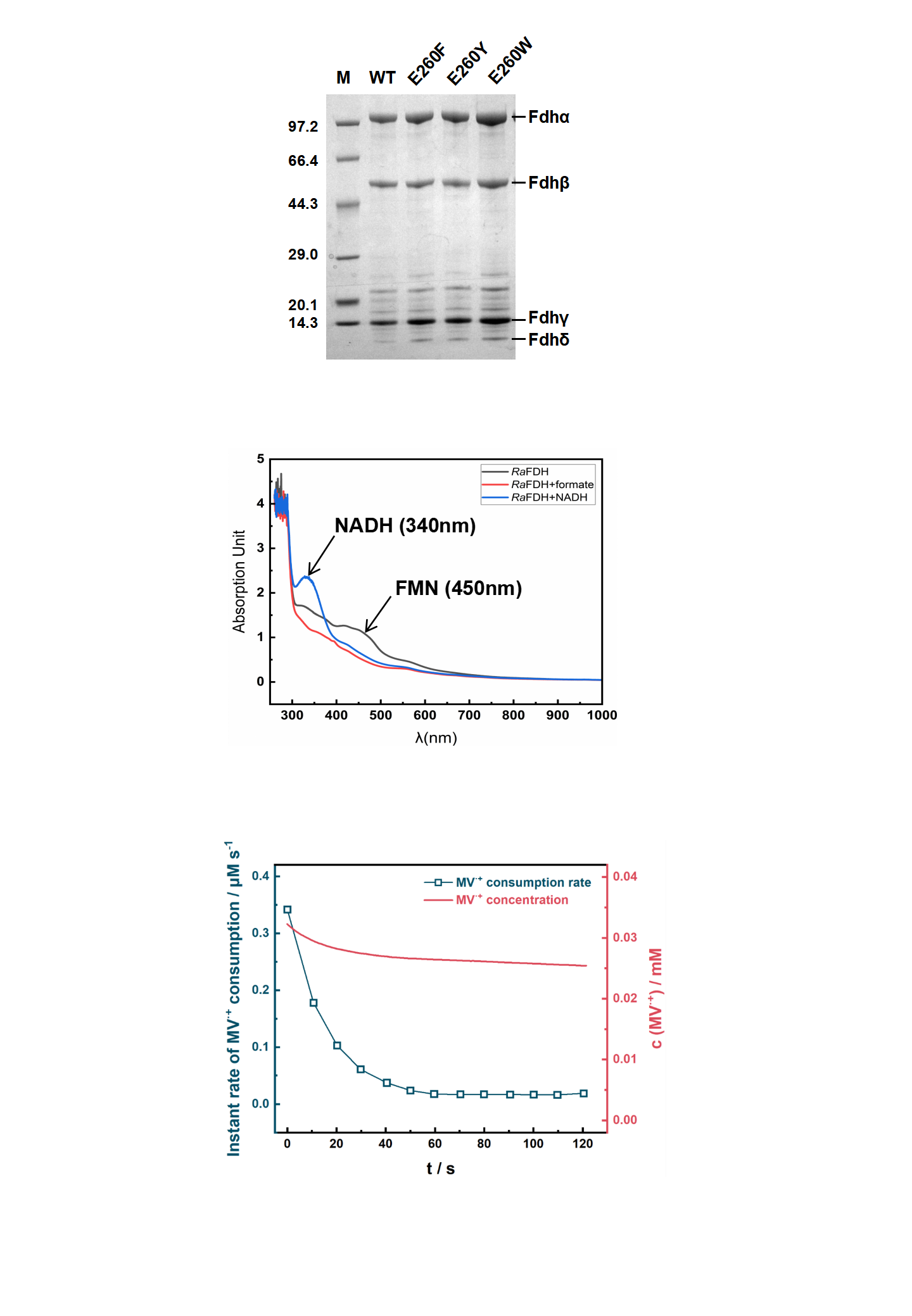


Figure S10. SDS-PAGE analysis of purified *Ra*FDH βE260F, βE260Y, βE260W expressed in *E. coli* MC1061; M: standard protein marker.


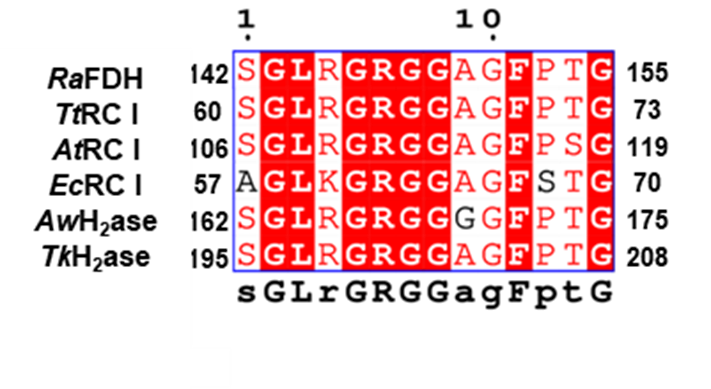
**
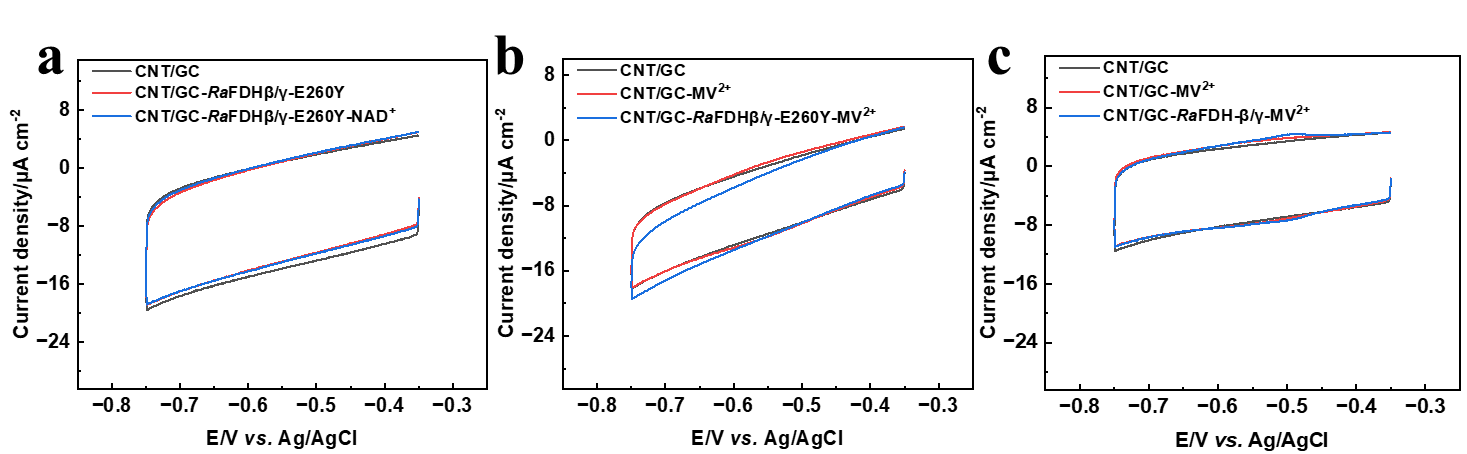
**Figure S11. Bioelectrocatalytic reduction of NAD^+^ and MV^2+^ by *Ra*FDH-β/γ and it’s E260Y variant.

Figure S12. Multiple sequence alignment of the key loop in NADH dehydrogenase homologues. from formate dehydrogenases of *Rhodobacter aestuarii* (*Ra*FDH), respiratory complex I from *Thermus thermophilus* (*Tt*RC I), *Arabidopsis thaliana* (*At*RC I) and *Escherichia coli* (*Ec*RC I), Hydrogenase from *Acetobacterium woodii* (*Aw*H2ase) and *Thermoanaerobacter kivui* (*Tk*H2ase), with residues color coded to highlight similarity.

Table S1. Primers used in this study.

| **Primer** | **Sequence (5´-3´)** |
| --- | --- |
| P1 | ATCTGTGTGGGCACTCGACCGG |
| P2 | CTACTGCCGCCAGGCAAATTCTG |
| P3 | CTGCTCGATGACCTTTGTGATACGATG |
| P4 | CCATCATCACCGCCTGCGTCAC |
| P5 | GACATCACTTTGGAAGCGGCTTATTGCC |
| P6 | CGGAACGGTGACCTCAACACCATC |
| P7 | GACGATGACAAGATGAAAGACCTGATCATTCCCCCG |
| P8 | TCAGGTCTTTCATCTTGTCATCGTCATCATATAAATCCCGCATCC |
| P9 | CTCGAGGGTACCATATGGGAATTCGAAGCTTGGCTGTTTTGG |
| P10 | GTACCCTCGAGTCATTCGGCGGCCTCCCTCAGCACG |
| P11 | TGCGGCTTTGAAACCGCGATGCTGAACTCG |
| P12 | GGTTTCAAAGCCGCAGACATAGGCCCCCG |
| P13 | TGCGGCTATGAAACCGCGATGCTGAAC |
| P14 | GGTTTCATAGCCGCAGACATAGGCCCC |
| P15 | TGCGGCTGGGAAACCGCGATGCTGAACTCG |
| P16 | GGTTTCCCAGCCGCAGACATAGGCCCCCGC |
